# The neurobiology of placebo effects in sports: EEG frontal alpha asymmetry increases in response to a placebo ergogenic aid

**DOI:** 10.1038/s41598-019-38828-9

**Published:** 2019-02-20

**Authors:** Ellen K. Broelz, Paul Enck, Andreas M. Niess, Patrick Schneeweiss, Sebastian Wolf, Katja Weimer

**Affiliations:** 1 University Hospital Tuebingen, Department of Psychosomatic Medicine and Psychotherapy, Osianderstr. 5, 72076 Tuebingen, Germany; 2 University Hospital Tuebingen, Department of Sports Medicine, Hoppe-Seyler-Str. 6, 72076 Tuebingen, Germany; 3 University of Tuebingen, Department of Psychology, Schleichstr. 4, 72076 Tuebingen, Germany; 4 Ulm University Medical Center, Department of Psychosomatic Medicine and Psychotherapy, Albert-Einstein-Allee 23, 89081 Ulm, Germany

## Abstract

The performance enhancing (ergogenic) placebo effect is elicited by an inert treatment and caused by positive affective appraisal of effort perception. Frontal alpha asymmetry (FAA) is a neurobiological correlate of positive affect. This study investigates, whether receiving an ergogenic placebo increases FAA and whether scores on the behavioral inhibition and activation system (BIS/BAS) scales affect this increase in FAA. Nineteen competitive male cyclists (37.26 ± 9.82 years) performed two maximum effort time trials. The first served as baseline for the second intervention time trial, where athletes received a placebo ergogenic aid or no treatment. We recorded FAA using EEG throughout all time trials and assessed BIS/BAS by questionnaire. There was a significant difference in change from baseline to intervention time trial in FAA during cycling in response to the placebo ergogenic aid compared to the control group. BIS, the BAS subscale Drive and the BAS-BIS difference score significantly co-varied with the change in FAA from baseline to intervention time trial in response to the placebo ergogenic aid. Administering a placebo ergogenic aid significantly influenced FAA during maximum effort cycling. Those athletes with a more pronounced goal seeking persistence and an overall dominance of the BAS over the BIS showed a significantly greater increase in FAA in response to a placebo ergogenic aid. A more pronounced BIS, however, seems to antagonize the increase in FAA associated with the ergogenic placebo response.

## Introduction

The placebo effect is defined as “any improvement in a symptom or physiological condition of individuals following a placebo treatment”^1^ (p.201). Accordingly, the ergogenic placebo effect (EPE) is the performance enhancing effect elicited by a placebo ergogenic aid (PEA). More specifically, the EPE is operationalized as performance enhancement without the expected increase in perceived exertion^2,3^ or even a decrease in fatigue^4^. Since the perception of effort is an unpleasant sensation, we assume that the EPE is associated with more positive affect. Moreover, a PEA increases reward probability by inducing expectations of performance enhancement and therefore functions as a positive affective cue.

The EPE has been shown in cycling using caffeine^5–7^ and carbohydrates^8^, and in running using sodium bicarbonate^9^ and a hypothetical new ergogenic aid^10^. In strength training, the EPE has been shown using anabolic steroids^11,12^, amino acids^13^ and caffeine^14^ in different compound and isolation movements such as bench press, leg extension and deadlift. A review of 12 studies found overall changes in performance ranging from −7.8% to +50.7% after ergogenic placebo interventions^15^. A meta-analysis of 14 studies reported small to moderate effects of placebo treatments in sports performance^16^. According to a recent survey based study of 79 elite athletes from different sports, 47% of elite athletes have experienced placebo effects first hand, the majority believe in the possible ergogenic effect of placebos (82%) and those with past placebo experiences were more likely to endorse a wider use of placebos in the sport setting than those without past placebo experiences^17^. Despite increasing interest in the brain mechanisms mediating the placebo response in motor performance^4,18–20^, to this day there is no study investigating cortical processes involved in the ergogenic placebo response (EPR) during high intensity endurance performance. This gap in research is caused by the fact, that most brain imagery methods are highly susceptible to artifacts due to movement and muscle contractions. However, technological developments in recent years have enabled the recording of cortical activation during endurance performance using active EEG electrodes, which amplify the electrical signals recorded from the scalp directly at the electrode, making it less susceptible to external electrical activity and movement artifacts^21,22^. Standardized test conditions on a bicycle ergometer further minimize the influence of artifacts and enable the recording of cortical parameters during exercise^23^. We therefore used EEG in this study of the EPR, as it allows the recording of brain activity during high intensity cycling, where the body is stationary while under high physiological stress.

EEG activity in the alpha frequency band (8–13 Hz) is inversely related to activation of the underlying cortex^24,25^ and builds the basis of the valence motivation model^26^. This model associates dominant left relative to right frontal activation, also known as frontal alpha asymmetry (FAA), with an approach motivational system and positive affect^27^. More specifically, the right prefrontal cortex is considered to be specialized for negative emotions and the left prefrontal cortex for positive emotions independent of the individual disposition and therefore represents a current affective state^28^. The EEG-affect-exercise relationship was also studied by Petruzzello and colleagues, who found that greater relative left frontal activation pre-exercise predicted increased positive affect and reduced anxiety post-exercise in those athletes engaging in high intensity exercise.

Asymmetric electrical activity in the prefrontal cortex has also been linked to trait motivational orientation^29^, which can be measured using the behavioral inhibition (BIS) and activation system (BAS) questionnaire^30^. A more pronounced BAS has been shown to make an individual highly responsive to reward cues, reflects the disposition to pursue goals and is positively associated with relatively more activation in the left than the right prefrontal cortex (higher FAA)^31^. The BAS has been divided into three subscales: BAS Drive reflecting the persistent pursuit of desired goals, BAS Fun reflecting a desire for new rewards and to approach potentially rewarding events, BAS Reward reflecting a positive responses to or anticipation of reward^30^. The BIS is hypothesized to be sensitive to signs of punishment and novelty in an attempt to control the experience of anxiety in response to anxiety-relevant cues, thus representing individuals with a more pronounced inward orientation^30^. To determine individual differences in the relative dominance of the BAS over the BIS, the BAS-BIS difference score can be calculated^32^.

A meta-analysis of the relationship between FAA and BAS across different conditions found only a weak and inconsistent correlation between both measures^33^. Questioning the assumptions that frontal EEG asymmetry represents a trait variable, Coan *et al*. suggested an alternative model for the approach/withdrawal motivational model of frontal EEG asymmetry and emotion called the capability model. This model suggests more pronounced differences in frontal EEG asymmetries during emotionally salient situations indicating that individual differences in affective style are a function of innate capabilities and context^34^. This model was supported by a study finding a correlation of male college students’ EEG asymmetry with their BAS scores only in the presence of an attractive female experimenter, interpreting this result as evidence for the important role of context in engaging FAA in situations with high approach motivation^35^. Finally, recent research explored the direct relationship between the BIS/BAS trait component, FAA at rest and in response to emotional cues as a function of stimulus valence. They showed that higher BAS values explained an increase in FAA at rest and in experimental condition for positive cues, while higher BIS values marked a decrease in FAA at rest and in experimental condition in response to negative cues^28^.

Sensitivity of the BAS, specifically in the subcategory of fun and sensation seeking, in combination with contextual cues in the form of treatment descriptors has been shown to enhance placebo responding^36^. A study concerned with the effect of self-control on asymmetric frontal cortical activation found that approach-prone persons (high BAS) were more likely to show increased relative left frontal cortical activation (high FAA) after exercising self-control^37^. Maximal effort endurance performance requires an exceptionally high level of self-control and the BAS seems to play a role in placebo responsiveness as well as engaging FAA as a self-control mechanism. Therefore, we measured the BIS and BAS trait to explore whether it predicts the expected increase in FAA during maximum effort cycling elicited by the administration of a PEA.

The purpose of this study was to investigate FAA as a cortical mechanism involved in the EPR and explore whether the BIS/BAS personality trait predicts the degree of FAA increase. Therefore, we recorded FAA as an indicator of change in the motivational valence system, in response to the administration of a highly salient PEA during an isokinetic maximum effort cycling time trial. Specifically, we hypothesized, that the placebo group will show an increase in FAA from baseline to intervention time trial, while the control group will show no change in FAA and that high scores on the BAS questionnaire including its three main subscales (Drive, Fun and Reward) and low scores on the BIS questionnaire affect this increase in FAA.

## Materials and Methods

The methods described here show only minor deviations from our previously published research protocol^38^.

### Participants

**Figure 1:**
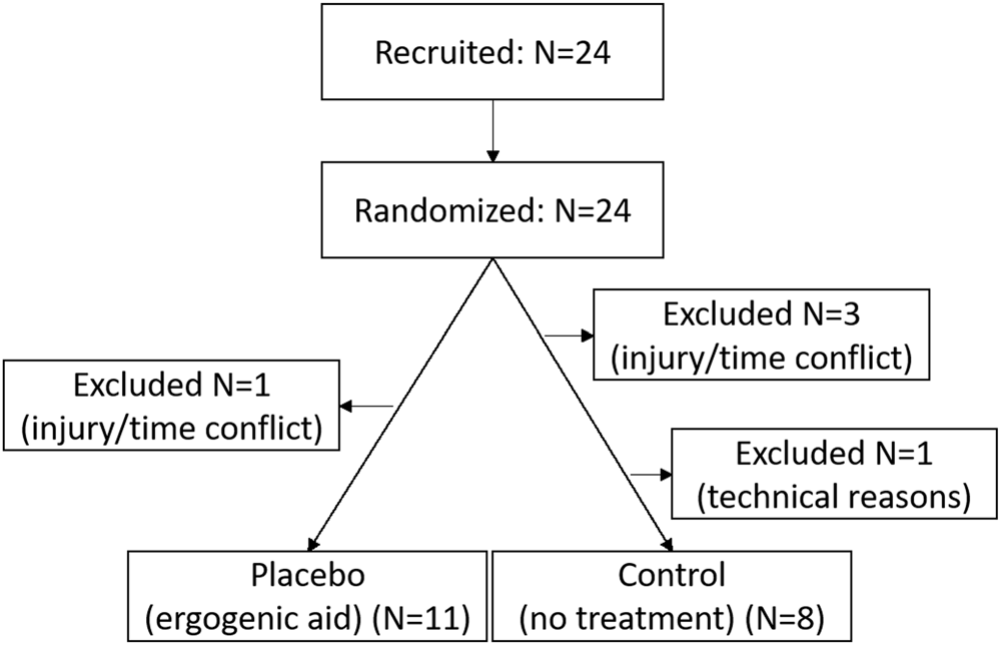
PRISMA Statement.

An a priori power analysis (G*Power Version 3.1.9.2.) for a 2 (group) × 2 (time) × 5 (block) within-between factors repeated measures ANOVA with an assumed effect size of Cohens’ f = 0.25 (power = 0.95, alpha = 0.05) revealed a required sample size of N = 20 participants. Taking possible drop-outs into account, we recruited 24 competitive cyclists (mountain bike, road bike or triathlon), of which four dropped out due to injury and timing conflicts, leaving 20 athletes to participate in the study. One participant had to be excluded from further analysis due to a technical recording defect, leaving 19 athletes with a mean age of 37.26 (SD = ±9.82) for further analysis (Fig. 1). All participants had to fulfill the following inclusion criteria: male, between 18 and 50 years of age, a minimum of 3 weekly training sessions during competition season and a minimum of 3 competitions per year. Exclusion criteria were serious illness, use of regular medication during the time of the study and a training pause in the 4 weeks immediately prior to the study. All data acquisition took place at the performance diagnostics laboratory at the Department of Sports Medicine at the University Hospital Tuebingen, Germany. The study was approved by the ethical committee of the University of Tuebingen (157/2015B01, 25.03.2015) and registered at the German Clinical Trials Register (Deutsches Register Klinischer Studien, DRKS, Study-ID: DRKS00008197, date of registration: 02.06.2015). All methods were applied according to the approved guidelines and regulations such as national legislation and the Declaration of Helsinki. All participants were enrolled after written informed consent only.

### Experimental procedure overview

Participants were block-randomized with a block size of N = 6 to receive either a placebo ergogenic aid (placebo group) or no treatment (control group) at the intervention time trial. Athletes visited the performance laboratory on three separate occasions. On the first visit their current training level was assessed, they were introduced to the EEG equipment and familiarized with the isokinetic cycling ergometer (SRM GmbH, Jülich, Germany). They performed a practice time trial to become familiar with the time trial protocol and to reduce learning effects, which could otherwise disguise changes from baseline (TT_b_) to intervention (TT_i_) time trial. After this session, participants were expected to be sufficiently familiar with the experimental set up. The following baseline and intervention time trials were held at the same time of day (8 am ± 1 h) and the same day of the week (Wednesday, Thursday or Friday) spaced 7 ± 1 days apart to control for performance differences based on daytime and weekday. Participants came to the laboratory fasted (12 h) and were not allowed to consume caffeine or nicotine prior to the time trials.

### Performance test and practice time trial

Upon arriving at the testing facility, athletes read the study description detailing all test sessions and the terms of their participation. After consenting, a medical doctor examined heart and lung function and cleared them for participation in the study. All subjects were tested using an ergometer based incremental performance test protocol, starting at a resistance of 40 Watt (W) and increasing by 40 W every 3 minutes. Participants cycled continuously until they were no longer able to maintain cadence above 65 revolutions per minute (rpm). Blood samples (20 µl) were taken from the right earlobe at rest and at the end of each 3 min interval to determine lactate concentration at each workload. The maximum lactate tolerance was determined using a stationary blood lactate analyser (Biosen S_line by EKF Diagnostics, Barleben, Germany) and the individual anaerobic threshold (IAT) was calculated using the Dickhuth method^39^.

After the performance test, subjects filled in the Behavioral Inhibition and Activation System questionnaire (BIS/BAS)^30,40^. The validation of the German version of the BIS/BAS questionnaire showed good to acceptable internal consistencies of the scales: BIS Cronbach’s α = 0.78, BAS α = 0.81, BAS drive α = 0.69, BAS fun α = 0.67, BAS reward α = 0.69^40^. Internal consistencies found were good to acceptable and comparable to reported ranges^40^ for BIS α = 0.68, BAS α = 0.84, BAS drive α = 0.74, BAS fun α = 0.75, but poor for BAS reward α = 0.49. The BAS-BIS difference score was calculated to determine individual differences in the relative strength of the BAS compared to the BIS by subtracting the z-transformed BIS score from the z-transformed BAS score for each participant in accordance with Sutton and Davidson (1997)^32^. After adjusting their cycling position on the ergometer, which was kept equal throughout all time trials, they performed a shortened version of the actual time trial procedure described below.

### Baseline and intervention time trials

**Figure 2:**
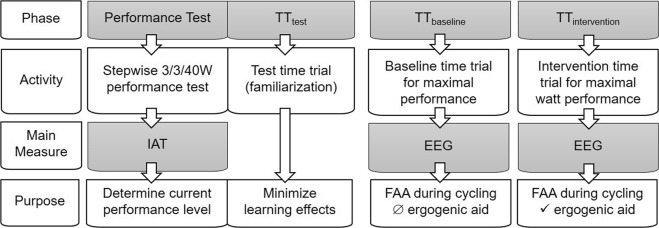
Study protocol detailing all laboratory visits including performance diagnostics to determine the individual anaerobic threshold (IAT), test time trial (TT_t_) as well as both baseline and intervention time trials (TT_b_ and TT_i_).

Upon arriving to their 2^nd^ and 3^rd^ laboratory session (TT_b_ and TT_i_), participants were set up with the EEG system. Subjects were instructed to drink 250 ml water during EEG setup. The protocol for TT_b_ and TT_i_ consisted of two warm up blocks (5 min each), five time trial blocks (9 min each) and one cool down block (5 min). An overview of all laboratory sessions is given in Fig. 2. We controlled for exercise duration, because a study had shown an inverted U-shaped dose-response relationship of FAA in response to increasing exercise durations, meaning an increase in FAA after medium length exercise duration (30 min) without changes for shorter (15 min) and longer (45 min) durations^41^. During both warm up and cool down, athletes cycled at varying cadence and a resistance of 1.5 W/kg bodymass. During the time trial blocks, they cycled at a fixed cadence of 95 rpm and aimed for maximal power output over time. We chose an isokinetic task at a fixed cadence, because previous research has shown, that variations in cadence may influence cortical activation evident in EEG recordings^42,43^. Specifically, athletes were instructed to go “all-out” and attempt to reach maximum effort in each time trial^44,45^. All 9 min blocks were preceded and followed by a 2 min rest period, during which participants reported their current RPE score, a lactate sample was taken, a 60 second EEG rest recording was taken in an upright seating position and athletes were allowed to drink water. All instructions for the time trial protocol were displayed on a computer screen set up on a tripod in front of the ergometer. During the pedalling blocks a countdown timer was displayed. At TT_b_, athletes received 250 ml water before and during the time trial, while at TT_i_ participants in the placebo group received 150 ml tab water to wash down 100 ml of a vanilla-grapefruit flavoured branched chain amino acid pudding, serving as a PEA, which has been shown previously to elicit an ergogenic placebo response in a 45 min maximum effort time trial^46^. In this study, we were able to demonstrate statistical equivalence between the active placebo (low dose BCAA) and its inert placebo counterpart. Athletes received an information sheet designed to increase expectancy associated with acute branched chain amino acid ingestion on high intensity endurance capacity, emphasizing its potential ergogenic properties. The exact expression was: “This study aims to investigate, whether branched chain amino acids can elicit an acute increase in endurance performance. The performance enhancing effect of these amino acids may be based on central as well as peripheral processes”. Cyclists in the control group received 250 ml water without an intervention. The precise amount of water was given to ensure equal stomach volume between groups. During the time trials all athletes were allowed to drink 250 ml of water in the designated time slots between blocks.

### EEG recording and data processing

At both time trials, 32 active electrodes set up in a flexible, breathable EEG cap (ActiCap, Brain Products GmbH, Gilching, Germany) were arranged in accordance with the international 10:20 system^47^ on the athlete’s skull. FCz was used as reference and AFz as ground. The active electrodes were filled with a high-viscosity electrolyte-gel for active electrodes (EASYCAP GmbH, Herrsching, Germany) to ensure optimal signal transduction with impedances below 10 kΩ. EEG raw data were amplified and recorded using the BrainAmp MR system and Vision Recorder at a sampling rate of 512 Hz and processed offline using BrainVision Analyzer 2.0 (Brain Products GmbH, Gilching, Germany). After down sampling to 256 Hz, butterworth zero phase high- and low-pass filters were applied (time constant 0.0318 s; 24 dB/octave), leaving a frequency range from 4 to 32 Hz, to eliminate drifts from sweat at very low frequencies and muscle artifacts above 40 Hz. Ocular correction was performed using a restricted infomax Independent Component Analysis^48^ based on a slope algorithm to detect eye blinks and eye movements from channels Fp1, Fp2 and Fpz. The detected eye blinks and movements were then separated and removed from the EEG recordings. After excluding the 4 electrodes very close to the neck musculature (TP9, PO9, PO10, TP10), a semi-automatic raw data inspection was used to detect artifacts, if the following criteria were not met: maximal voltage step of 50 µV/ms, maximal difference of 200 µV/20 ms interval, maximal amplitude between −200 µV and +200 µV and lowest activity 0.5 µV/100 ms interval. Subsequently, all data were visually inspected and marked artifacts were either confirmed or rejected. If a channel’s signal comprised >10% artifact contaminated intervals, its signal was topographically interpolated with data of its four nearest neighbors using interpolation by 4^th^ order spherical splines with a maximal degree of the Legendre polynomial of 10^49^. Then, data were baseline corrected and fragmented into data sets of 4-s duration. A Hanning Window (10%) was applied to all artifact-free epochs before a Fast Fourier Transformation was used to obtain the mean power areas for the alpha frequency band (8–13 Hz), which was then exported for further analysis.

The frontal alpha asymmetry score (FAA) was calculated by subtracting the natural log transformed alpha power in left frontal region (F3, F7, Fp1) from the natural log transformed power in right frontal region (F4, F8, Fp2)^50^.

### Statistical analysis

To test for group differences in terms of subject characteristics, we performed student’s t-tests. To analyze effects of receiving a PEA on FAA during exercise independent of exercise duration, a 2 (group: P, C) × 2 (time: TT_b_, TT_i_) × 5 (block: 9 min each) repeated measures ANOVA was applied. To test the influence of the trait variables behavioral inhibition system (BIS) and behavioral activation system (BAS) as well as their subscales (BAS drive, BAS reward and BAS fun) and the BAS-BIS difference score on the effect of the PEA on FAA, we calculated multiple 2 (group: P, C) × 2 (time: TT_b_, TT_i_) repeated measures ANCOVAs for mean FAA with the trait variables as covariate: separate ANCOVAs for each trait variable as the BAS total score and subscales were significantly intercorrelated. Main effects and interactions were reported and the effect size (η^2^) was calculated. Post-hoc *t-*tests were used to specify significant results and Cohen’s d was calculated. The level of significance was set at *p* ≤ 0.05. All data were analyzed using SPSS version 19.0 (IBM SPSS Statistics for Windows, Armonk, NY, USA) for Windows.

## Results

### Sample characteristics

There were no differences in age (*t*(17) = 1.51, *p* = 0.15), training level (IAT) (*t*(17) = 1.12, *p* = 0.28), maximal lactate tolerance (*t*(17) = 1.80, *p* = 0.09), training frequency (*t*(17) = −1.51, *p* = 0.15) and Body Mass Index (*t*(17) = 0.26, *p* = 0.80) between groups (Table 1).

**Table 1:** Sample characteristics. Descriptive statistics are shown as mean value ± standard deviation. Group differences are calculated using t-tests.

	Placebo	Control	Statistic
No. of participants	11	8	
Age (years)	34.5 ± 9.7	41.1 ± 9.2	*p* = 0.15
IAT (W)	208.6 ± 25.9	223.0 ± 30.1	*p* = 0.28
Max. lactate tolerance (mmol)	11.5 ± 3.1	13.8 ± 1.8	*p* = 0.09
Training frequency (h/week)	9.5 ± 3.2	7.3 ± 2.9	*p* = 0.15
Body Mass Index (kg/m²)	23.2 ± 2.4	23.5 ± 1.6	*p* = 0.80

### Changes in FAA

**Figure 3:**
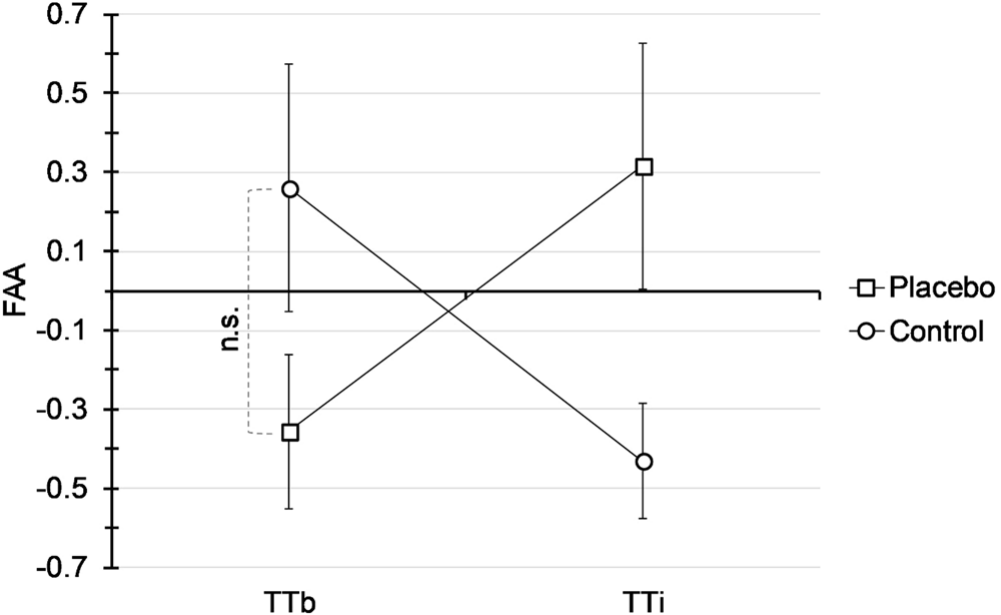
Mean change in FAA presented as the natural logarithm of alpha power (ln *α* µV²) recorded from the left frontal cortex (F3, F7, Fp1) subtracted from the right frontal cortex (F4, F8, Fp2) over 45 minutes (5 blocks of 9 minute each) (see text for details). Error bars show standard deviation. The repeated measures ANOVA revealed a significant interaction of time and group (*F*(1,17) = 5.56, *p* = 0.03, *partial η*^2^ = 0.25). Post-hoc t-test for TT_b_ was not significant.

The repeated measures ANOVA to show the influence of the PEA on FAA revealed a significant interaction of time and group (*F*(1,17) = 5.56, *p* = 0.03, *partial η*^2^ = 0.25) (Fig. 3). There was no significant interaction of time, block and group (F(4,68) = 0.95, *p* = 0.44), time and block (*F*(4,68) = 1.09, *p* = 0.37), or block and group (*F*(4,68) = 0.63, *p* = 0.64). There was no significant main effect of time (*F*(1,17) = 0.001, *p* = 0.98) or block (*F*(4,68) = 0.60, *p* = 0.67). T-tests showed no significant difference in mean FAA between groups at TT_b_ (*t*(17) = 1.73, *p* = 0.10, *d* = 0.78), and a significant difference in ΔFAA between groups (*t*(17) = −2.36, *p* = 0.03, *d* = 1.13).

### BIS/BAS trait

**Figure 4:**
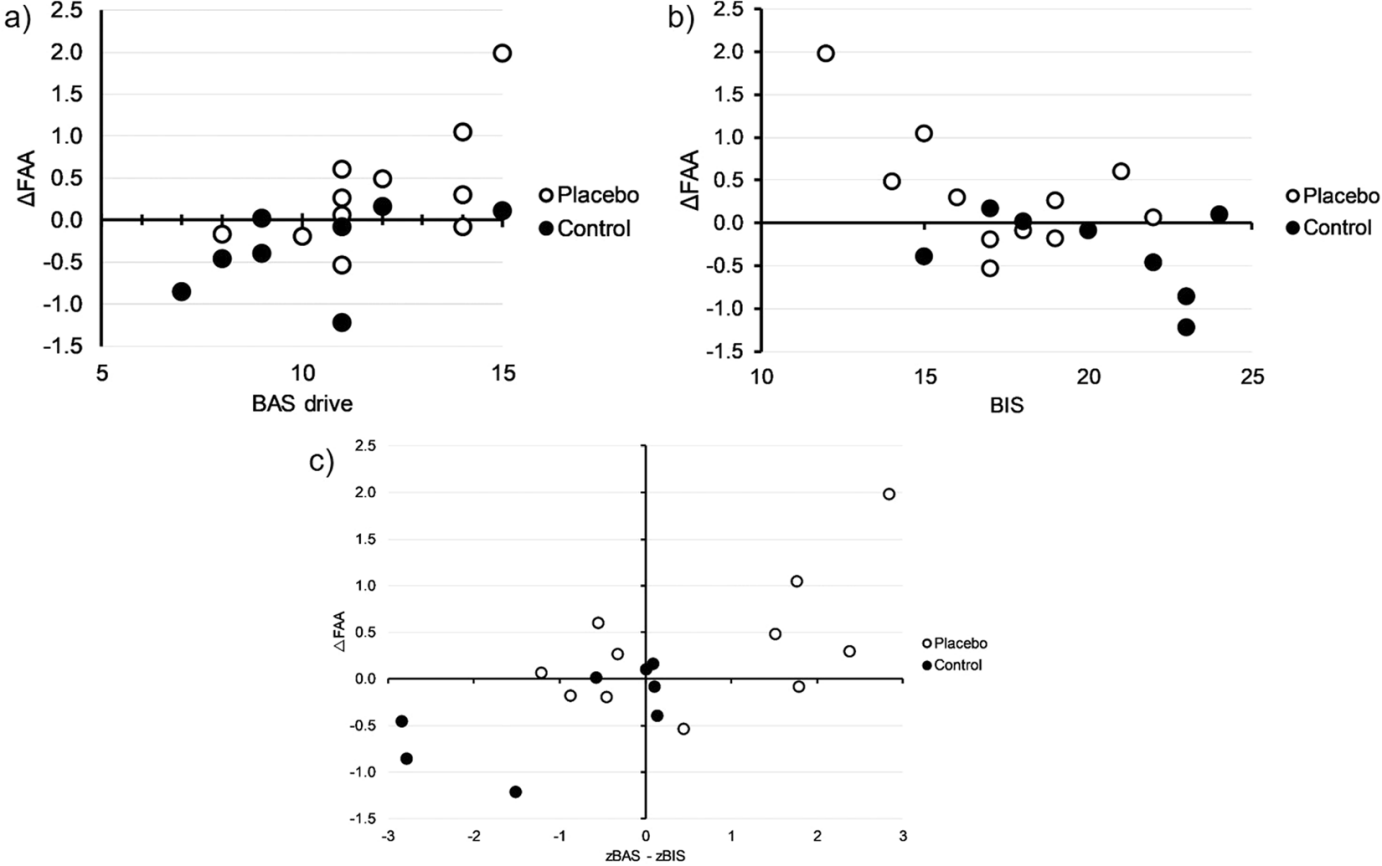
Correlation between BAS Drive, BIS and BAS-BIS difference with change in FAA. Repeated measures ANCOVAs showing the influence of the trait variable (**a**) BAS drive, (**b**) BIS and (**c**) BAS-BIS difference on the change in frontal alpha asymmetry from TT_b_ to TT_i_ (Δ FAA) in response to a placebo ergogenic aid compared to a control group.

Independent of group, there were significant positive correlations between ΔFAA and BAS (r = 0.48, p = 0.04), ΔFAA and the BAS subscale Drive (r = 0.63, r = 0.004) and ΔFAA and the BAS-BIS difference score (r = 0.70, p = 0.001), but no significant correlations between FAA and the BAS subscales Fun (r = 0.25, p = 0.31) and Reward (r = 0.32, p = 0.18). Furthermore, there was a significant negative correlation of BIS and ΔFAA (r = −0.61, p = 0.005). The repeated measures ANCOVAs to show the influence of the BIS and BAS trait variables on the effect of the PEA on ΔFAA revealed a significant interaction of time and BIS (F(1,16) = 5.44, p = 0.03, *partial η*^2^ = 0.25) (Fig. 4b), time and the BAS-BIS difference score (F(1,16) = 8.95, p = 0.01, *partial η*^2^ = 0.36) (Fig. 4c) as well as the BAS subscale Drive (F(1,16) = 7.21, p = 0.02, *partial η*^2^ = 0.31) (Fig. 4a). There were no significant interactions of time and BAS (F(1,16) = 2.62, p = 0.13), BAS Fun (F(1,16) = 0.32, p = 0.58) and BAS Reward (F(1,16) = 0.77, p = 0.39).

## Discussion

To investigate a cortical mechanism involved in the ergogenic placebo response in competitive cyclists, we recorded EEG during high intensity all-out time trials on an isokinetic ergometer and measured the change in FAA in response to a PEA. We also measured the BIS/BAS trait to analyze the degree to which it is associated with these changes in FAA. The main findings of this study indicate, that athletes who received a placebo ergogenic aid showed an increase in FAA during a cycling time trial compared to athletes who received no intervention. Further, there was a significant correlation between the BAS score and the change in FAA across all athletes, showing the validity of our measure. Lastly, those athletes with a more pronounced persistence in the pursuit of desired goals (BAS subscale Drive) were more likely to show an increase in relative left frontal cortical activation (FAA) and those athletes with a more pronounced sensitivity for punishment, nonreward and novelty (BIS) were more likely to show a decrease in FAA in response to a placebo ergogenic aid compared to those athletes who received no intervention (Fig. 4a,b).

Our results are in line with the valence motivation model of FAA, suggesting that cortical hemispheric differences are a result of positive or negative valence of emotional conditions representing a current affective state^28^. A PEA can be considered a positive affective cue, because its administration induces high expectations by increasing reward probability in the form of performance enhancement. According to the valence motivation model, the ergogenic placebo response would then act by a positive situational affective appraisal. Although our results show a significant interaction of time and group for FAA, it is important to note, that this interaction is not solely driven by the increase in FAA in the placebo group, but also by a decrease in FAA in the control group (Fig. 3). This is likely due to a nocebo effect elicited by the disappointment of being randomized to the control group, which may indirectly cause negative expectations.

Certain personality traits are thought to interact with environmental cues and thereby influence neurobiological responses to treatments, indicating their role in the modulation of the placebo response. In the past, extraversion has been linked to placebo responding in irritable bowel syndrome patients^51^, trait optimism and anxiety were shown to predict placebo and nocebo effects in analgesia^52,53^ and dopaminergic function associated traits like novelty seeking, harm avoidance, behavioral drive, fun seeking and reward responsiveness have been linked with placebo analgesia^54^. A recent study showed, that a composite of three positive (ego-resiliency, altruism, straight-forwardness) and one negative (angry hostility) personality trait predicted 25% of placebo analgesia responding^55^. The same authors also tested several other personality traits, among them the behavioral inhibition and activation system (BIS/BAS), unfortunately however, they did not report the results.

To the best of our knowledge, the influence of personality traits in the context of ergogenic placebo responding has not been investigated so far. We measured the BIS and BAS trait and the subscales BAS Drive, BAS Fun and BAS Reward as well as the BAS-BIS difference score to assess the degree to which it affects the change in FAA. As expected, we found a significant positive correlation between BAS and change in FAA, BAS-BIS difference and change in FAA and a significant negative correlation between BIS and change in FAA across all athletes. BIS, the subscale BAS Drive and the BAS-BIS difference score significantly co-varied with the interaction of time and FAA. This indicates that a more pronounced persistence in the pursuit of desired goals in athletes facilitates the EPR as measured by state changes in FAA (Fig. 4a), while a more pronounced BIS, which inhibits behavior leading to possible negative outcomes, antagonizes the EPR (Fig. 4b). Finally, a dominance in BAS over BIS also facilitated the EPR (Fig. 4c).

However, our results did not show the expected influence of the overall BAS trait in the cortical response to a specific cue (here the administration of a PEA). Our results are therefore only partially in line with a recent study exploring the direct relationship between the BIS/BAS trait, lateralized frontal brain activation at rest and in response to emotional cues. Using regression analysis, Balconi *et al*. were able to show a significant predictive role of BIS/BAS sensitivity in explaining the lateralized cortical activation in response to positive and negative emotional cues^28^. Future research could expand on our results by stratifying athletes based on their BIS/BAS scores or the BAS-BIS difference scores.

Many researchers have attempted to find a “placebo personality” marking the difference between those who do and those who do not respond to placebo treatments^56^. Our results are partially in line with the view that different personality dispositions might respond to different contextual cues. More recently, Darragh *et al*. suggests, that individuals with an outward oriented approach behavioral style are more likely to respond to placebo treatments, which are novel, reward and goal oriented, externally focused and accompanied by positive interaction^57^. A review by Horing *et al*. identified several predictors for placebo responders, among them the psychological construct of emotional valence attached to the event, including BAS related constructs such as goal seeking, self-efficacy, optimism and locus of control^58^. In a motion sickness paradigm of placebo responding, generalized self-efficacy, an internal locus of control and cognitive flexibility predicted symptom improvement in the placebo compared to the control group^59^. We were able to show, that the same is true for athletes scoring high on the BAS subscale Drive, low on the BIS scale or showed BAS dominance over BIS (high BAS-BIS difference score) and receiving a novel tasting PEA, which they were told has the potential to increase their endurance performance. Our findings also fit in with the capability model proposed by Coan *et al*., suggesting more pronounced differences in frontal EEG asymmetries during emotionally salient situations indicating that individual differences in affective style are a function of innate capabilities and context^34^. Considering BIS/BAS sensitivity to be a valid measure of affective style and the administration of a PEA to be a salient emotional cue, because it indicates reward probability, in accordance with this model, the PEA is more readily processed by individuals with high persistence in the pursuit of desired goals, therefore resulting in a larger FAA increase. Specifically, those individuals with a match between their individual response system and the environmental cue show higher responsiveness to a placebo ergogenic aid and those with a mismatch show lower responsiveness. We employed a novel placebo (highly salient grapefruit vanilla pudding), accompanied by goal and reward oriented instructions (endurance performance enhancement) and external focus (maximizing power output) and found higher response rates in those individuals who show stamina in following their goals (BAS Drive) (Fig. 4a) and lower response rates in those who show inhibitory tendencies in the pursuit of goals (BIS) (Fig. 4b). This pattern has been shown specifically for placebo responding, where individuals with higher BAS sensitivity showed a greater placebo effect in response to an outwardly oriented treatment^36^.

Recognizing the high level of self-control at play in an all-out cycling time trial, our results are further supported by a recent study concerned with the effect of self-control on asymmetric frontal cortical activation. The authors referred to self-control as “the capacity to override or alter a predominant response tendency” (p.282)^37^, which is clearly the case in a maximal effort endurance task, where the predominant response tendency would be to stop cycling in order to reduce the sensation of effort and discomfort. The study by Schmeichel *et al*. tested the influence of temporary self-control on FAA in response to affective cues and found that approach-prone persons (high BAS) are more likely to show increased relative left frontal cortical activation (high FAA) after exercising self-control especially in response to positive valence emotional cues^37^. More specifically, they found, that those subjects who scored high on BAS and low on BIS (high BAS-BIS difference score) showed increases in FAA and a low BAS-BIS difference (relative strength of the BIS over the BAS) predicted a reduction in FAA. These findings are in line with our results showing an overall positive correlation between BAS-BIS difference score and ΔFAA as well as an interaction of time and the BAS-BIS difference score, indicating antagonistic effects on placebo responding in those individuals who are more withdrawal motivated (Fig. 4c).

The present study is limited mainly by study design constraints due to the novelty of recording EEG during high intensity endurance exercise. We did not include power output, lactate production and RPE in the analysis, because our study design was specific to the necessities of EEG recording during exercise. The study was designed with repeated interruptions to allow for EEG rest recordings, in case of problems with noise and artefacts during high intensity pedaling. Fortunately, the quality of data during cycling was high. However, these interleafed breaks did not allow for linear lactate accumulation over time and caused a ceiling effect with the RPE ratings. It is also likely, that the differences in FAA between groups would have been more pronounced in a continuous cycling time trial. Also, we cannot exclude the possibility that the small and non-significant baseline difference in FAA between groups may have contributed to a type-I error. Lastly, the sample size was relatively small and therefore the results of this study need to be confirmed by future studies investigating larger sample sizes.

Despite these limitations, this study enabled a novel approach to investigate the neurophysiological link between placebo-induced expectation and endurance performance using an objective measure (EEG). While most previous studies have looked at exercise related cortical parameter by recording brain activity before or after endurance exercise or during single joint movements, we investigated the cortical correlates of placebo modulation during a high intensity endurance task for the first time.

## Conclusion

To the best of our knowledge, this is the first study, which investigated a cortical mechanism involved in the administration of a PEA in a maximal effort cycling time trial. The aim of the present research was to test the increase in FAA in response to a PEA and explore the role of the behavioural activation and inhibition system (BAS/BIS) including its subscales Drive, Fun and Reward as well as the BAS-BIS difference score as trait variables able to predict changes in FAA.

According to our results, the administration of a novel, highly salient PEA may indeed lead to changes in cortical processing, specifically increased FAA compared to a control group. Athletes with a more pronounced BAS Drive and a less pronounced BIS trait appear more likely to respond to the administration of a placebo ergogenic aid with an increase in FAA, thus suggesting high persistence in the pursuit of desired goals and low sensitivity to signals of punishment and nonreward as traits mediating the EPR.

It has long been established, that positive feelings during a task reliably predict future involvement in that specific activity^60^. Therefore, PEAs could be applied to enhance sports motivation and exercise adherence by increasing enjoyment and positive affect during high intensity training. Nonetheless, these results are based on competitive cyclists and require further confirmation in health oriented and rehabilitation training.

Future studies should look at changes in FAA in response to placebo ergogenic aids during a continuous time trial without interleafed pauses, since we showed, that recording EEG activity during vigorous cycling is possible. Employing a continuous time trial design would allow for a translation of the increase in FAA in response to a PEA to a behavioral outcome containing subjective (RPE), physiological (lactate) and objective (power output) measures of effort.

## Data Availability

The datasets generated and analyzed during this study are available from the corresponding author upon reasonable request.
